# Bibliographical Notices

**Published:** 1840-05-23

**Authors:** 


### BIBLIOGRAPHICAL NOTICES.

*The Salt Sulphur Spring of Virginia.* By THOMAS D. MÜTTER, M. D. Philadelphia, 1840.

Dr. MÜTTER visited the Virginia Springs in 1834 and 1839, and while there, "having been, from a variety of causes, forced into a very extensive practice," he is induced to make public what he had collected relative to the therapeutic influence of the different springs. He informs us, that he proposes to devote a pamphlet to each of these waters, and commences in the one before us with the "Salt Sulphur." Dr. Mütter evidently writes more for the general than the professional reader, for "the fashionable man and the victim of disease," rather than the physician, and cannot therefore claim a very extended notice of his brochure at our hands. He has given us a pleasant and detailed description of the Salt Sulphur spring, including situation, scenery, means of access, "accommodations, table, and bar,"—with the diseases to which it is applicable, the proper mode of its employment, and a long list of certificates to the curative properties of the waters, as well as to "the unwearied attentions of the host, and satisfactory arrangements of his establishment." To the visiter to the Virginia Springs, Dr. Mütter's pamphlet will be an invaluable vade mecum—which, we have no doubt, does nothing more than justice to the waters of the Salt Sulphur, and the hospitable host who dispenses them.

*Transactions of the Medical Society of the State of New York.* Vol. IV.—Part III.

The volume of the Transactions of the Medical Society of New York, just published, is replete with useful and interesting information. The *Statistics of Medical Colleges* is a paper of great value, both for present use and future reference. Dr. Hull's address on improvement in medicine, Dr. Purple's address on professional duties, and the report of a committee of the society on medical education, are well written pieces, presenting interesting views on topics that repeated discussion has rendered somewhat threadbare. A lengthy and elaborate essay of Dr. Davis on diseases of the spinal column, and a rare case, reported by Dr. M'Naughton, which we have marked for future insertion, make up the present highly interesting volume of transactions.

